# Current practices and evaluation of barriers and facilitators to surgical site infection prevention measures in Jimma, Ethiopia

**DOI:** 10.1017/ash.2021.227

**Published:** 2021-11-17

**Authors:** Leigh R. Berman, Andrew Lang, Beshea Gelana, Samuel Starke, Dawd Siraj, Daniel Yilma, Daniel Shirley

**Affiliations:** 1 University of Wisconsin School of Medicine and Public Health, Madison, Wisconsin; 2 University of Wisconsin Health, Madison, Wisconsin; 3 Department of Health Policy and Management, Jimma University, Jimma, Ethiopia; 4 Division of Infectious Disease, Department of Medicine, University of Wisconsin School of Medicine and Public Health, Madison, Wisconsin; 5 Department of Internal Medicine, Jimma University, Jimma, Ethiopia; 6 Jimma University Clinical Trial Unit, Jimma University, Jimma, Ethiopia

## Abstract

**Objective:**

Surgical site infections (SSIs) greatly burden healthcare systems around the world, particularly in low- and middle-income countries. We sought to employ the Systems Engineering Initiative for Patient Safety (SEIPS) model to better characterize SSI prevention practices and factors affecting adherence to prevention guidelines at Jimma University Medical Center (JUMC).

**Design:**

Our cross-sectional study consisted of semistructured interviews designed to elicit perceptions of and barriers and facilitators to SSI prevention among surgical staff and observations of current preoperative, perioperative, and postoperative SSI prevention practices in surgical cases. Interviews were recorded, manually transcribed, and thematically coded within the SEIPS framework. Trained observers recorded compliance with the World Health Organization’s SSI prevention recommendations.

**Setting:**

A tertiary-care hospital in Jimma, Ethiopia.

**Participants:**

Surgical nurses, surgeons, and anesthetists at JUMC.

**Results:**

Within 16 individual and group interviews, participants cited multiple barriers to SSI prevention including shortages of water and antiseptic materials, lack of clear SSI guidelines and training, minimal Infection Prevention Control (IPC) interaction with surgical staff, and poor SSI tracking. Observations from nineteen surgical cases revealed high compliance with antibiotic prophylaxis (94.7%), hand scrubbing (100%), sterile gloves and instrument use (100%), incision site sterilization (100%), and use of surgical safety checklist (94.7%) but lower compliance with preoperative bathing (26.3%), MRSA screening (0%), and pre- and postoperative glucose (0%, 10.5%) and temperature (57.9%, 47.3%) monitoring.

**Conclusions:**

Utilizing the SEIPS model helped identify institution-specific barriers and facilitators that can inform targeted interventions to increase compliance with currently underperformed SSI prevention practices at JUMC.

Surgical site infections (SSI), though largely preventable, contribute to increased patient morbidity and mortality, length hospital stays, cost of healthcare, and antimicrobial resistance.^1,2^ Although SSIs pose significant challenges for hospitals globally, the burden of SSIs is significantly greater in low- and middle-income countries (LMICs) compared to high-income countries.^3^ In Ethiopia specifically, several single-center studies have reported SSI rates as high as 10%–20%, 5–10 times greater than estimated rates in the United States.^4–9^ Both the World Health Organization (WHO) and the Center for Disease Control have provided evidence-based SSI prevention guidelines consisting of 20–30 practices demonstrated to reduce rates of SSI, but significant challenges to widespread implementation of these practices exist in LMICs, such as Ethiopia.^10,11^

Successful implementation of SSI prevention strategies requires multimodal interventions tailored to institution-specific strengths and weaknesses, rather than one-size-fits-all guidelines.^12^ The Clean Cut Programme, conducted at 5 hospitals in Ethiopia and piloted at Jimma University Medical Center (JUMC), adopted this mentality by engaging local stakeholders in adaptive process mapping to implement site-specific interventions and improve compliance to 6 SSI prevention practices.^4,13,14^ Although Clean Cut’s results and efforts to address institution-specific implementation barriers are promising, the disconnect between the high estimated SSI rates and few studies informing successful implementation and sustainability of guidelines in LMICs highlights the need for further research in this area.^15^

The Systems Engineering Initiative for Patient Safety (SEIPS) serves as a valuable quality improvement tool by highlighting the complex interactions between key elements of the work system—person, tools and technology, organization, environment, and task—that cumulatively shape patient care processes and drive patient and institutional outcomes, both desirable and undesirable.^16–18^ We utilized the SEIPS framework to better characterize SSI prevention practices and factors affecting adherence to prevention guidelines at JUMC over time. With this foundation, we hoped to identify future interventions for improving effectiveness and sustainability of SSI prevention strategies. This project is part of a larger collaboration between JUMC and the University of Wisconsin (UW). Previous work has included identifying barriers and facilitators to Infection Prevention Control (IPC) team establishment and hand hygiene among healthcare workers (HCWs) at JUMC.^19^

## Methods

### Study setting and design

Our mixed-methods, cross-sectional study took place over a 4-week period in March 2019 at JUMC, a tertiary-care hospital in southwestern Ethiopia. As one of the largest and oldest teaching hospitals in Ethiopia, JUMC serves a catchment population of >15 million and ∼1,800 obstetric procedures, 3,000 elective nonobstetric procedures, and 3,000 emergency operations are performed there each year. JUMC has a functioning IPC Team and Patient Safety Committee established in 2018.

### Interviews

We conducted semistructured interviews with surgical nurses, surgeons, and anesthetists at JUMC. HCWs included in the study worked in the main operating room (OR) or the maternity OR and were able to converse in either English or Amharic. Participants were selected via convenience sampling, but efforts were made to interview individuals from a variety of professional categories. An interview guide including open- and closed-ended questions was developed within the SEIPS framework to elicit current SSI practices as well as barriers and facilitators to SSI prevention (Appendix 1). Literature review revealed a minimum of 12 interviews would attain theoretical saturation.^20^ All interviews were recorded, manually transcribed, and thematically coded using QRS Nvivo (version 12.4.0) within the 5 SEIPS elements of the work system. Quotations were further characterized as “facilitators,” “barriers,” “attitudes,” and/or “current practices.” Barriers were subcoded as either “easy to modify” or “difficult to modify.” Coding was performed primarily by 1 investigator (LB) with frequent review and discussion with another (DS) to ensure accuracy and reliability. Barriers and facilitators were then ranked by frequency of discrete quotations.

### Observations

Trained data collectors followed surgical patients throughout their pre-, peri-, and postoperative care to determine whether recommended SSI prevention practices occurred. Compliance to safe surgical practices was recorded using an observation checklist written in English and designed by study staff using 2018 WHO and UW Health SSI prevention tools (Appendix 2). The observation form enabled observers to mark completion of recommended SSI prevention tasks and add relevant notes about hospital and HCW practices. Surgeries within the main OR and maternity OR were selected for observation via convenience sampling, but we included a variety of emergency and elective procedures. Completed standardized observational forms were collected and recorded in Microsoft Excel (Microsoft, Redmond, WA) to determine rates of adherence.

### Ethical considerations

Ethical approval was obtained from both Jimma University and the University of Wisconsin Institutional Review Boards. Verbal consent was obtained from participants prior to interviews.

## Results

We conducted a total of 16 interviews with 20 JUMC participants; 15 interviews involved 1 participant and 1 group interview involved 5 participants. Participants included OR nurses (40%), surgeons (50%), and nurse anesthetists (10%). Moreover, 65% of participants worked primarily in the major OR, 15% worked primarily in the maternity OR, and 20% worked in both. The remaining demographic information of the participants is outlined in Table 1. Trained data collectors observed 19 surgeries, of which 9 took place in the maternity OR and 10 in the major OR.

**Table 1.** Interview Participant Demographics (N = 20)

Characteristic	Nurses (N = 8)	Surgeons (N = 10)	Anesthetists (N = 2)
**Primary unit**			
Major OR, no. (%)	6 (75)	6 (60)	1 (50)
Maternity OR, no. (%)	2 (25)	1 (10)	0 (0)
Both, no. (%)	0 (0)	3 (30)	1 (50)
**Sex**			
Male, no. (%)	7 (87.5)	10 (100)	2 (100)
Female, no. (%)	1 (12.5)	0 (0)	0 (0)
Age, median y	25.5	34.5	28.0
Median experience in current position, y	2	6	5
Median number of surgeries per day^a^	4	1	4

Note. OR, operating room.

a Assumes 5-d work week.

### Perceived and observed adherence to safe surgery practices

Perceived and observed compliance with safe surgery practices were assessed using patient interviews and perioperative observations by trained data collectors respectively (Table 2 and Supplementary Table 1). In interviews, participants stated that methicillin-resistant *Staphylococcus aureus* (MRSA) screening (which is not directly included in the WHO SSI prevention guidelines, though the WHO does recommend decolonization of *S. aureus* carriers prior to many types of surgeries to reduce rates of SSI) and bathing were rarely performed at JUMC.^10^ Participants in most interviews stated antibiotics were consistently given 30–60 minutes before incision for nonemergency cases (75%), with ceftriaxone alone noted as the most common antibiotic given. Although interviewees reported patients are usually required to purchase the antibiotics on their own, patients in the maternity OR are occasionally provided antibiotics if they cannot afford them (12.5%). Interviewees provided varying reports of whether and how hair is removed prior to surgery. Participants in 56.3% of interviews agreed hair was rarely removed in the OR, but others described hair removal using clippers (6.25%), razors (25.0%), and scissors (6.25%). A strong majority of participants stated that sterile gloves and instruments are routinely used (100%), that surgeons consistently scrub with soap and water (93.8%), and that surgical sites are cleaned with povidone prior to surgery (62.5%) or that alcohol is used if povidone is unavailable (100%). Participants in 50.0% of interviews stated that surgeons commonly write postoperative wound care orders and instructions.

**Table 2.** Reported Adherence and Observed Compliance With SSI Prevention Measures

SSI Prevention Measure	Reported Adherence, From Interviews (No. of Comments, N = 16 Interviews)	Observed Compliance, From Data Collectors, No. (% of total)
**Preoperative**		
MRSA screening	Not done (16)	0 (0.0)
Pre-op bathing	At home maybe (2) In-wards sometimes (4) No (10)	5 (26.3)
Antibiotic timing^a^	30–60 min (12), At time of surgery for emergencies (4)	18 (94.8) within 120 min of surgery
Antibiotic choice	Ceftriaxone (13) Metronidazole for certain procedures (4)	
Hair not removed or removed with clippers^b^	Not removed at hospital (9)—appropriate Done by clipper in OR (1)—appropriate Done by razor in OR (4) Done by scissors in OR (1) Done at home (3)	17 (89.5)
**Intraoperative**		
Surgeon scrubs with soap and water OR alcohol scrub^a^	Usually soap/water (15) then alcohol based on availability (13)	19 100)
Surgeon uses sterile gloves^a^	Yes (16)	19 100)
Instruments documented sterile^a^	Yes (16)	19 100)
Incision site skin prep in OR^a^	Povidone iodine (10) Alcohol if iodine runs out (16) Sometimes have to use normal saline only (1)	19 (100)
**Postoperative**		
Wound care order submitted	Surgeon writes order (8) Not done (4)	19 (100)

Note. SSI, surgical site infection; OR, operating room.

a Core component of the Clean Cut Initiative.

b WHO Guidelines recommend hair removal should generally NOT be performed prior to surgery, and if performed, should only be removed with dedicated sterile clippers. Shaving is strongly discouraged.

Observations revealed preoperative bathing (observed in 26.3% of surgeries), pre- and postoperative glucose monitoring (0%, 10.5%), mechanical bowel preparation (5.3%), MRSA screening (0%) were infrequently performed. Pre-, intra-, and postoperative temperature monitoring were variably performed in 57.9%, 73.7%, and 47.3% of surgeries respectively. We observed high rates of compliance with antibiotic prophylaxis (94.7%), hand scrubbing (100%), use of sterile gloves (100%), use of sterility indicators (100%), sterilization of the incision site (100%), maintenance of sterility throughout the procedure (100%), fraction of inspired oxygen kept ≥50% (100%), and use of the surgical safety checklist (94.7%). Although the WHO recommends against removing hair preoperatively or, if absolutely necessary, using clippers, inappropriate hair removal occurred in a small number of observed cases (10.5%).^10^

### Barriers and facilitators

**Fig 1.**
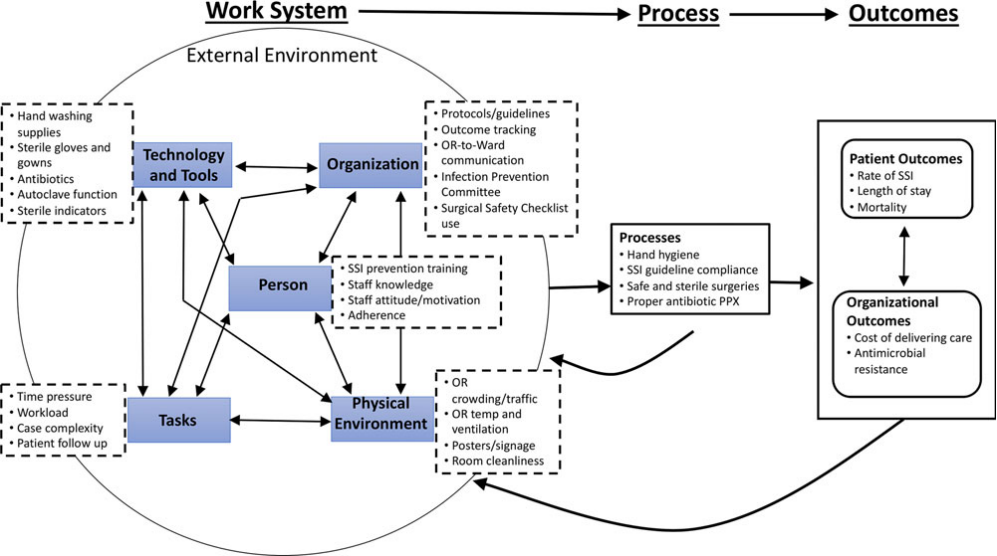
Systems Engineering Initiative for Patient Safety (SEIPS) diagram for SSI prevention at Jimma University Medical Center, March 2019. Within this modified SEIPS diagram, dashed boxes list common themes identified in interviews, and the circle depicts a work system of inter-related elements (double headed arrows) that cumulatively shape downstream patient and organizational outcomes (left-to-right arrows).^18^ The model accounts for adaptability within the system whereby process evaluation and outcome monitoring can feedback to identify and strengthen vulnerabilities within the system (right-to-left arrows).

To better understand gaps in implementation of SSI prevention practices, we categorized interview responses within the SEIPS framework (Table 3 and Fig. 1). Also, 543 interview excerpts were coded to 1 or more of the SEIPS elements. Among them, 350 (64%) were deemed barriers and 193 (36%) were deemed facilitators. Representative quotes for identified barriers and facilitators are included in Table 4.

**Table 3.** Barriers and Facilitators to SSI Prevention Characterized Within the SEIPS model and Ranked by the Number of Times Each Theme was Cumulatively Mentioned Within All Interviews

Tools and Technology	Organization	Environment	Person	Tasks
Barriers				
Insufficient antiseptic (26), water (25), or soap (10)	Lack of protocols and guidelines (30)^a^	High OR traffic (14)	Insufficient training on SSI prevention (38)^a^	Lack of patient follow-up (28)
Inadequate supply of gloves (20)	IP staff uninvolved (12)^a^	Poor OR ventilation (7)	Staff not following protocols (10)	Time pressure in emergency cases (17)
Lack of antibiotic choice (9)	Poor communication between OR and ward (6)	Too hot in the OR (5)	Poor staff attitude or motivation (2)	High workload (9)
Shortage of cleaning tools (4)	Minimal tracking of patient outcomes (4)	Poor OR zone signage (5)^a^		Complicated surgeries (8)
Cultures not available (4)	Teaching hospital setting (2)	Few bathrooms in hospital (3)		Communication about antibiotics (8)
Facilitators				
Sterile instrument indicators (23)	Culture of speaking up about breaks in sterility (16)	Environmental services disinfects room and table (6)	Staff knowledgeable about SSI prevention (14)	Surgeries are generally short (1)
Available water and antiseptics (11)	Infection Prevention staff known (9)	OR is separate space (5)	Motivation to prevent SSI (6)	Good wound care (1)
Antibiotics on hand (5)	Informal notification about surgical complications (7)	Hospital is new (4)	Good training in IP (6)	Rational use of antibiotics (1)
Sufficient gloves (4)	SSC (5)	Hospital is clean (3)	Good training (2)	
Waste and sharps containers (3)	National/International guidelines known (3)	Handwashing posters are present (3)		
Central supply (2)				

Note. SSC, surgical safety checklist; SSI, surgical site infection; IP, infection prevention; SEIPS, Systems Engineering Initiative for Patient Safety.

a Characterized as easy-to-modify, relative to other barriers.

**Table 4.** Representative quotes developed from interview responses within the SEIPS* model.

Code	Theme	Representative Quote
**Tools and technology**		
Barrier	Shortages of water, antiseptics, and gloves	“Most of the time, the supplies. No supplies most of the times. Maybe the gloves. Some gloves have powder and when we are not trying to glove, it may be not out.”
Facilitator	Instrument indicators	“There are indicators. They were autoclaved and the indicators are put externally and they change color. The way to see externally if it is dirty is observation. Both the indicators and observation.”
**Organization**		
Barrier	Lack of IPC presence	“Why I said no, if there is an [IPC] as a figure, if they don’t work together with us working in the OR, it is not right to say there is an infection prevention team. And also…does not communicate with surgeons.”
Facilitator	SSI prevention checklist	“There is an infection prevention protocol. The protocol paper usually the scrub nurse will fill the protocol before starting the operation. Oh, a checklist? We draw the checklist. But we do not always have it”
Facilitator	Informal communication between wards and surgery	“Usually, we will communicate. If for example, I did the surgery and the patient has an infection and if she was diagnosed on the ward, he will tell him.”
**Person**		
Barrier	Lack of training	“One important thing this infection prevention skills should be given or trained for the residents, for the nurses, and for the internists. There should be training. I stayed in this hospital for four years. Even a single day, nothing for infection prevention. But for all the residents and the internists and nurses, before they go to the OR room, they should have basic training at least techniques of scrubbing”
Facilitator	Motivation to prevent SSIs	“It is my duty. My responsibility…. should observe all the medical students, the residents, the surgeons, or someone else around. Should be observed for one who is contaminating or breaking sterility. If there is one who is breaking sterility you should tell him, you are contaminated.”
**Task**		
Barrier	Emergency cases	“There are many emergency cases. I mean, it is the only one performing these surgeries…it is the catchment population for more than 15 million, you know, surgically speaking…so many difficulties come with.”
Barrier	Limited SSI tracking	“Yeah actually, this is difficult because the majority of them do not come back unless they have a major complication. For minor complaints, they will not come back. So, we don’t know how many percent of them develop surgical site infection really.”
**Environment**		
Barrier	OR crowding and open doors	“Most of the time, the difficulty is that the doors should always be self- closing… If it is opened, one is forgetting to close and other people get into the OR… Most of the time, you are on surgery, when you turn about, someone is inside in the OR. There is a new person.”
Facilitator	Effective environmental service workers	“There are the cleaners. The cleaners are hard workers. Usually after transporting one patient from the table, they clean the room.”

Note. SEIPS, Systems Engineering Initiative for Patient Safety; OR, operating room; IPC, infection prevention and control.

### Tools and technology

Participants overwhelmingly cited shortages of antiseptics, clean water, gloves, soap, and antibiotics as barriers to SSI prevention. Several participants noted that povidone iodine is particularly prone to shortages. One participant stated that when the hospital “run[s] out of alcohol and iodine, we may be forced to use only normal saline at times.” Additionally, multiple interviewees discussed that preoperative MRSA screening is not widely available at JUMC due to resource limitations. Serving as facilitators, multiple participants stated that antiseptics, antibiotics, and gloves are more often available than not, and sterile instrument indicators are consistently available (Table 4, quote 2).

### Organization

Organizational barriers included lack of clear SSI prevention guidelines and minimal formal communication routes between surgery and ward staff, making coordination of tasks like preoperative bathing and tracking postoperative outcomes challenging. Although there is a functional IPC team at JUMC, participants reported limited interaction between the IPC team and surgical staff (Table 4, quote 3). Lack of hospital tracking of patient outcomes was also discussed. According to one participant: “The chances of nurses would identify those surgical site infections and include them in the data of the … that it might be surgical site infection [data] is not that complete.”

An important organizational facilitator was positive patient safety culture at JUMC because participants stated they feel comfortable and obligated to speak up about sterility breaks. Participants also highlighted the use of the standardized, perioperative Surgical Safety Checklist (SSC) introduced by the Clean Cut Programme, although participants stated the checklist forms were not always readily available (Table 4, quote 4).

### Person

Lack of SSI prevention training for HCWs and students involved in surgery was unanimously cited by interview participants. Most participants felt they were knowledgeable about SSI but advocated for formal training for all new surgical students and staff (Table 4, quote 6). Major facilitators included staff attitudes toward perceived effectiveness of perioperative standards in preventing SSI and individual motivation for patient safety (Table 4, quote 7).

### Task

As task barriers, interview participants cited the difficulty of SSI tracking and long-term patient follow-up, and they noted that patients come from large geographic distances and usually do not return to JUMC unless a major complication occurs. Participants conveyed a perceived lack of time to perform SSI prevention tasks in emergency cases and a sentiment that high workload impedes compliance with SSI prevention practices. Multiple interviewees also described perceptions of increased risk of SSI in complicated patients. As one participant stated, “People usually come here in late presentation, with an infection that is already complicated and advanced. So surgical site infection and deeper wound infection are also very common.” Task-related facilitators included statements that surgeries are commonly short and antibiotic use is appropriate and rational.

### Environment

Environmental barriers included crowding and traffic in the ORs given frequent presence of students, poor ventilation, minimal cooling mechanisms, and lack of clear zoning of sterile spaces. As environmental facilitators, participants stated the ORs are adequately isolated from the rest of the hospital and kept clean by effective environmental services staff.

## Discussion

Overall, we found high compliance with the specific surgical practices targeted by the Clean Cut Programme, with variably lower adherence to other WHO recommended practices. Our observational findings aligned with Clean Cut 2018 post-intervention data at JUMC showing 60%–100% compliance to preoperative antibiotic prophylaxis, surgical scrubbing, sterile glove use, incision site antisepsis, and use of sterile instrument indicators.^14^ Measures not included in Clean Cut but often included in SSI prevention bundles such as preoperative bathing, glucose and temperature monitoring, and discontinuation of antibiotics within 24 hours were performed less consistently. Our study found high congruence between perceived and observed practices which may reflect HCW understanding of and adherence to SSI prevention strategies emphasized in the past and currently considered routine at JUMC. Notably diverging from this congruence, only a handful of interview participants reported that surgical staff commonly use the Surgical Safety Checklist and submit postoperative wound care orders, but these tasks were observed in 94.7% and 100% of surgeries, respectively. These few instances of incongruence may be due to observer bias leading to increased attention to these tasks.

Our finding that ceftriaxone alone was commonly used for preoperative antibiotic prophylaxis matches other reports from Ethiopia.^21,22^ A 2019 report from a government hospital in southern Ethiopia found that ceftriaxone reasonably covers most pathogens implicated in SSIs from clean procedures based on local susceptibility patterns, though first-generation cephalosporins, such as cephazolin, are generally preferred for most procedures to reduce antimicrobial resistance.^8,23,24^ Unfortunately, several studies from Ethiopia suggest first-generation cephalosporins are less widely available than ceftriaxone.^23,24^ Because of growing antibiotic resistance in Ethiopia, rational antibiotic use should be a priority for the JUMC IPC, and further studies are needed to determine factors that contribute to antibiotic choice at JUMC including availability of antibiotic options and local antimicrobial resistance patterns.^25^

High estimated rates of SSI at JUMC and specific gaps in preoperative bathing, glucose and temperature monitoring, SSI checklist usage, and discontinuation of antibiotics within 24 hours demonstrate opportunities for additional intervention.^4,9^ To our knowledge, this is the first study using the SEIPS model to systematically examine barriers and facilitators to SSI prevention in a LMIC, tertiary hospital. Visualizing the interacting elements within the SEIPS model (Fig. 1) allowed for identification of site-specific, multimodal interventions to improve SSI prevention (Table 5).

**Table 5.** Multimodal interventions to improve SSI prevention adherence at JUMC.

• Clarification and dissemination of institutional SSI prevention guidelines (O, P, T) • Formal SSI prevention training for new OR staff (O, P, T) • Recruitment OR staff to collaborate on the IPC team (O, P, TT) • Increased signage in OR spaces to improve zoning and encourage SSI prevention steps (E, P) • Enhanced SSI tracking and reporting (P, T, Pr, Ou)
Note. SSI, surgical site infection; E, environment; P, person; O, organization; T, task; TT, tools and technologies; Pr, processes; Ou, outcomes.

As similarly described in Clean Cut’s qualitative assessment of barriers to SSI prevention at other hospitals in Ethiopia, limited availability of water, soap, antiseptics, and gloves affects multiple WHO recommended SSI prevention practices including scrubbing, incision site disinfection, double gloving, and preoperative bathing.^15^ Although the IPC program and hospital can work to establish a locally produced source of antiseptic solution, the realities of resource limitations at JUMC must shape the IPC’s educational and organizational interventions. When antiseptics are available, the IPC team should organize workflow systems to ensure that HCWs have access to those materials. When antiseptics are limited, surgical staff should be made aware of the preferred method of hand hygiene as well as appropriate alternatives. Improved access to soap and water should be a priority.

Although interview results show knowledge of SSI as a facilitator, the variability of several SSI prevention practices demonstrates the need for enhanced SSI training and establishment of standardized SSI processes. Standardization will hopefully increase awareness of underused but proven SSI prevention strategies and allow for more standardized surveillance of bundle measure compliance by IPC staff. Improvements in SSI prevention knowledge via increased training and clear guidelines will likely modestly improve adherence to SSI prevention measures, but studies show improvements in infection control behaviors are more likely to be sustained with a multimodal implementation approach that incorporates principles of behavioral theory such as feedback, incentives, and social and leadership influence.^26–30^

To create feedback and incentive programs and enhance social influence to improve SSI prevention practices, the IPC should focus on a few possible additional interventions based on key barriers and facilitators discussed in interviews. First, increased IPC visibility within operating rooms and the hospital at large will provide leadership on SSI prevention. The IPC could consider designating willing surgical staff as ‘IPC champions,’ tasked with regularly discussing SSI prevention with staff and thereby promoting social influence over individual and group behaviors.^30^ Second, regular IPC monitoring of SSI prevention practices would enable surgical staff to receive feedback on their adherence rates. Consistent reporting of major and maternity OR compliance with standardized SSI practices may create friendly, intrafacility competition, which has been shown to increase compliance with other infection prevention practices such as hand hygiene.^31^ For countries like Ethiopia, without national reporting and penalties for high SSI rates and low IPC measure compliance, intrafacility competition may be an incentive for adherence feedback to influence SSI practices. Lastly, enhanced SSI tracking and reporting would provide incentives for HCWs to improve compliance, given that individual motivation to prevent poor outcomes was a facilitator discussed in interviews and other studies have suggested availability of outcome data encourages infection control behaviors.^29,30^ Several factors make SSI tracking challenging at JUMC including lack of patient follow-up, limited formal communication between surgical and ward teams, and lack of accurate recording of SSI in patient charts.4 Educational or organizational interventions aimed at increasing recognition and recording of SSI for both surgical and ward staff and developing systems that enable the IPC to gather and routinely report SSI data may begin to tackle these challenges.

Beyond strategies to improve implementation of existing SSI recommendations, our interviews revealed several additional steps the IPC can take to make SSI prevention easier at JUMC. Interview participants indicated that clear zoning of sterile OR areas, limiting OR traffic, and purchasing powder-free gloves may encourage better SSI prevention.

Our study has several limitations. Because our interview participants were all chosen via convenience sampling among those who agreed to take part in a study about SSI prevention, interview results were subject to selection bias and may not be representative of the entire JUMC workforce. Interview results are also subject to response and interviewer bias, particularly the small group interview where group and social dynamics may have influenced participants’ responses. Given the single-center design of our study, these results may not be representative of other hospitals in Ethiopia or other LMICs.

Despite its limitations, our study demonstrated the utility of the SEIPS model in examining factors affecting SSI prevention in underresourced healthcare settings and identifying areas for improvement. WHO guidelines and the Clean Cut Programme outline effective strategies for reducing SSIs; however, these tools cannot address all barriers within a given institution, and significant challenges to their implementation and sustainability exist. The SEIPS model can be used to understand specific implementation gaps and to guide future interventions based on pre-existing hospital strengths to ensure sustained improvement.

## Supplementary material

For supplementary material accompanying this paper visit https://doi.org/10.1017/ash.2021.227. click here to view supplementary material
