# Supplementary material for: Current practices and evaluation of barriers and facilitators to surgical site infection prevention measures in Jimma, Ethiopia
Source: Antimicrob Steward Healthc Epidemiol. 2021 Nov 17;1(1):e51. doi: 10.1017/ash.2021.227 (PMC9495540; doi:10.1017/ash.2021.227)
Supplement: Supplementary file 1 [file S2732494X21002278sup001.docx]

| **Supplemental Table 1.** Observed compliance with SSI prevention standards by OR location. | | | |
| --- | --- | --- | --- |
| Surgical Site Infection Prevention Measure | All observations  N (%) | Maternity OR  N (%) | Main OR  N (%) |
| Total Participants | 19 | 9 | 10 |
| *Preoperative* |  |  |  |
| - MRSA screening | 0 (0.0) | 0 (0.0) | 0 (0.0) |
| - Pre-op bathing, night before | 4 (21.0) | 1 (11.1) | 3 (30.0) |
| - Pre-op bathing, morning of | 1 (5.2) | 0 (0.0) | 1 (10.0) |
| - Antibiotics given within 120 min of surgery ^a, b^ | 18 (94.8) | 9 (100) | 9 (90.0) |
| - Appropriate hair removal ^c^ | 17 (89.5) | 8 (88.9) | 9 (90.0) |
| - Patient temp taken | 11 (57.9) | 4 (44.4) | 7 (70.0) |
| *Intraoperative* |  |  |  |
| - Surgeon scrubs with soap and water ^a^ | 19 (100) | 9 (100) | 10 (100) |
| - Surgeon uses sterile gloves ^a^ | 19 (100) | 9 (100) | 10 (100) |
| - Instruments documented as sterile via indicator ^a^ | 19 (100) | 9 (100) | 10 (100) |
| - Incision site prep performed ^a^ | 19 (100) | 9 (100) | 10 (100) |
| - Sterility maintained through procedure ^a^ | 19 (100) | 9 (100) | 10 (100) |
| - Body temp checked and ≥36 | 14 (73.7) | 6 (66.7) | 8 (80.0) |
| - FiO2 monitored and ≥50% | 19 (100) | 9 (100) | 10 (100) |
| - OR closed during surgery | 18 (94.8) | 8 (88.9) | 10 (100) |
| - Surgical Safety Checklist used ^a^ | 18 (94.8) | 9 (100) | 9 (90.0) |
| *Post-Operative* |  |  |  |
| - Body temp checked and ≥36 | 9 (47.4) | 3 (33.3) | 6 (60.0) |
| - Operative antibiotics discontinued within 24 hr | 15 (78.9) | 6 (66.7) | 9 (90.0) |
| - Wound covered with sterile dressing for at least 24hr | 19 (100) | 9 (100) | 10 (100) |
| - Wound care order submitted | 19 (100) | 9 (100) | 10 (100) |
| - OT hard surfaces cleaned after surgery | 19 (100) | 9 (100) | 10 (100) |
| ^a^ Measures targeted by the Clean Cut Programme  ^b^ Antibiotic choices: for Main OT, 9 of 9 used Ceftriaxone; for Maternity OT, 8 of 9 used Ceftriaxone alone, 1 used Ceftriaxone + Metronidazole  ^c^ Appropriate hair removal defined as hair not removed OR removed pre-operatively using a buzzer | | | |
